# Patient-specific anatomical model for deep brain stimulation based on 7 Tesla MRI

**DOI:** 10.1371/journal.pone.0201469

**Published:** 2018-08-22

**Authors:** Yuval Duchin, Reuben R. Shamir, Remi Patriat, Jinyoung Kim, Jerrold L. Vitek, Guillermo Sapiro, Noam Harel

**Affiliations:** 1 Center for Magnetic Resonance Research, Department of Radiology, University of Minnesota, Minneapolis, MN, United States of America; 2 Surgical Information Sciences, Minneapolis, MN, United States of America; 3 Departments of Electrical & Computer Engineering, Computer Science, Biomedical Engineering and Math, Duke University, Durham, NC, United States of America; 4 Department of Neurology, University of Minnesota, Minneapolis, MN, United States of America; Oslo Universitetssykehus, NORWAY

## Abstract

**Objective:**

Deep brain stimulation (DBS) requires accurate localization of the anatomical target structure, and the precise placement of the DBS electrode within it. Ultra-high field 7 Tesla (T) MR images can be utilized to create patient-specific anatomical 3D models of the subthalamic nuclei (STN) to enhance pre-surgical DBS targeting as well as post-surgical visualization of the DBS lead position and orientation. We validated the accuracy of the 7T imaging-based patient-specific model of the STN and measured the variability of the location and dimensions across movement disorder patients.

**Methods:**

72 patients who underwent DBS surgery were scanned preoperatively on 7T MRI. Segmentations and 3D volume rendering of the STN were generated for all patients. For 21 STN-DBS cases, microelectrode recording (MER) was used to validate the segmentation. For 12 cases, we computed the correlation between the overlap of the STN and volume of tissue activated (VTA) and the monopolar review for a further validation of the model’s accuracy and its clinical relevancy.

**Results:**

We successfully reconstructed and visualized the STN in all patients. Significant variability was found across individuals regarding the location of the STN center of mass as well as its volume, length, depth and width. Significant correlations were found between MER and the 7T imaging-based model of the STN (r = 0.86) and VTA-STN overlap and the monopolar review outcome (r = 0.61).

**Conclusion:**

The results suggest that an accurate visualization and localization of a patient-specific 3D model of the STN can be generated based on 7T MRI. The imaging-based 7T MRI STN model was validated using MER and patient’s clinical outcomes. The significant variability observed in the STN location and shape based on a large number of patients emphasizes the importance of an accurate direct visualization of the STN for DBS targeting. An accurate STN localization can facilitate postoperative stimulation parameters for optimized patient outcome.

## Introduction

Identification of subcortical brain structures for deep brain stimulation (DBS) surgery on standard clinical magnetic resonance images (MRI) is challenging and may lead to inaccurate targeting [1]. Suboptimal electrode placement has been associated with reduced efficacy and adverse effects [2,3]. One possible reason for the inaccurate targeting is that standard clinical MRI protocols at 1.5 Tesla (T) or 3T are associated with low resolution and relatively low signal to noise ratio (SNR). As a result, it is difficult to differentiate between the subthalamic nucleus (STN) and the adjacent substantia nigra (SN), to distinguish between the internal and external segments of the globus pallidus (GP), or to separate the nuclei within the thalamus. Therefore, most DBS surgeries trajectory plans rely on indirect and/or a combination of direct and indirect targeting methods. Indirect targeting incorporates normalized atlas-derived diagrams or some form of consensus coordinates that are superimposed on the patient’s own head MRI scan to approximate target location that is then modified based on visualization of subcortical landmarks structures [4]. However, the significant anatomical variability that exists between individuals, together with poor visualization of subcortical structures and their borders used for direct targeting, often lead to targeting errors [3,5,6].

High-field 7T MRI provides enhanced SNR and high-resolution images with an improved contrast and visualization of brain structures that are otherwise unobservable in-vivo [7]. Duchin et al. [8], characterized the amount of geometrical distortion present at 7T relative to standard clinical imaging obtained on a 1.5T scanner, in subjects undergoing preoperative evaluations for DBS surgery. They have demonstrated that at the center of the brain and in the midbrain region, there is minimal distortion when comparing the 7T with 1.5T images [8]. Thus, they concluded that, similar to standard 1.5T images that are being used for stereotactic planning, 7T images can be utilized for clinical applications. In this work, we demonstrate the ability, based on 7T MRI data, to visualize and create a patient-specific 3D anatomical model of the STN and validate the model against neurophysiological and clinical data obtained from the same patients. The data demonstrate the variability of the STN location and shape across patients. We further suggest that the development of ultra-high field instrumentation, combined with an assortment of image post-processing and visualization approaches, have progressed to a point where direct clinical applications can now be pursued. Results reported here support the use of 7T images for a) better localization and targeting of the STN; b) accurate localization of the final location of the DBS electrode and its stimulation contacts relative to the STN; and c) provide a framework to refine current DBS approaches, including determining the optimal location, stimulation settings and development of patient-specific strategies for treatment.

## Materials and methods

### Participants

72 patients (57 men and 15 women; average age 63.3±10.5 YO) were enrolled in this study. Of this group, 62 patients were diagnosed with Parkinson’s disease (PD) and 10 with Essential Tremor (ET). Out of the 62 patients diagnosed with PD, 54 underwent DBS of the STN and the GPi was targeted for the remaining 8. Essential Tremor patients that were scanned on the 7T MRI were added to this study although their target for DBS surgery was ventral intermediate nucleus (VIM). The ET patients have similar demographics to the PD patients and allow us to increase the sample size of the segmented STN. The study did not interfere or change the routine patients’ treatment protocol except for one extra 7T MRI scan. Subjects were recruited from the population of patients in the Department of Neurology of the University of Minnesota. Inclusion criteria included patients with a diagnosis of idiopathic Parkinson’s disease or Essential Tremor that were suitable candidates for DBS surgery [9]. The study was approved by the Institutional Review Board at the University of Minnesota (IRB #1210M22183) and all patients provided informed consent.

### Data acquisition

All subjects underwent preoperative scanning on a 7T MRI system, using T_1_-Weighted (T_1_W), T_2_-Weighted (T_2_W), and susceptibility-weighted (SWI) imaging. All patients were scanned with an actively shielded 7T magnet, using SC72 gradients capable of 70 mT/m and a 200 T/m/s slew rate, driven by a Siemens console (Erlangen, Germany). The 7T images were acquired with a 32-element head array coil (Nova Medical, Inc, Burlington, MA) and were acquired with the MRI vendor’s 3D distortion correction, which compensates for geometrical distortions originating from gradient nonlinearities.

All subjects also underwent standard clinical imaging on a clinical 1.5T or 3T MRI system prior to the 7T scan. Finally, a pre-operative computed tomography (CT) image, containing stereotactic head frame information, and a post-operative CT image were acquired. The former was acquired the day of the surgery while the latter was obtained several weeks after the surgery in order to ensure that potential brain shift resulting from the surgery is resolved before assessing final electrode location.

#### Acquisition parameters

T_1_-Weighted MRI: 3D acquisition, 0.6 mm isotropic resolution, FOV: 230 x 187 x 153 mm^3^ (312 x 384 x 256 matrix), TR = 3100 milliseconds (ms); TI = 1500 ms, TE = 3.5 ms, nominal flip angle = 6°, Total acquisition time = 6.5 min, acceleration factor of 2 (GRAPPA) along the right-left (RL) phase encode direction.

T_2_-Weighted MRI: An axial and coronal slab were acquired using a 2D turbo spin echo (TSE) sequence with the following image parameters: FOV: 200 x 200 x 26 mm^3^; 512 x 512 x 26 matrix (0.39 x 0.39 x 1.0 mm^3^), TR/TE 9000/58 ms, flip angle = 150°, acceleration factor of 3 (GRAPPA) along the anterior-posterior (AP) and RL phase encoding directions for axial and coronal orientation, respectively. The number of slices was optimized manually for each patient in order to maximize spatial coverage of the mid-brain while minimizing acquisition time. The total acquisition time was around 6 minutes for each T_2_-Weighted scan.

SWI-Acquisitions: 3D flow-compensated gradient echo sequences were acquired, using the following parameters: 60 slices, FOV = 200 x 200 x 48 mm^2^; 512 x 512 x 60 matrix (0.39 x 0.39 x 0.8 mm^3^), TR/TE = 28/21 ms; flip angle = 17°; bandwidth = 121 Hz/pixel; 6/8 partial Fourier; parallel imaging using an acceleration factor of 2 (GRAPPA) along the AP and RL phase-encoding direction for axial and coronal orientation, respectively. Acquisition time was 5 minutes for each SWI scan. The SWI sequence is a GRE sequence but for clarity and for applicability of readers replicating this work, we used the Siemens product name. SWI sequence is susceptible to off-resonance tissue/anatomical distortions. To mitigate for this we used an oblique slab to avoid air cavity and reduce susceptibility artifacts.

Note that the complete dataset was acquired with a protocol that allowed obtaining the necessary information in a clinical relevant acquisition time (total acquisition time of ~30 minutes) while minimizing motion artifacts.

### STN segmentation

**Fig 1:**
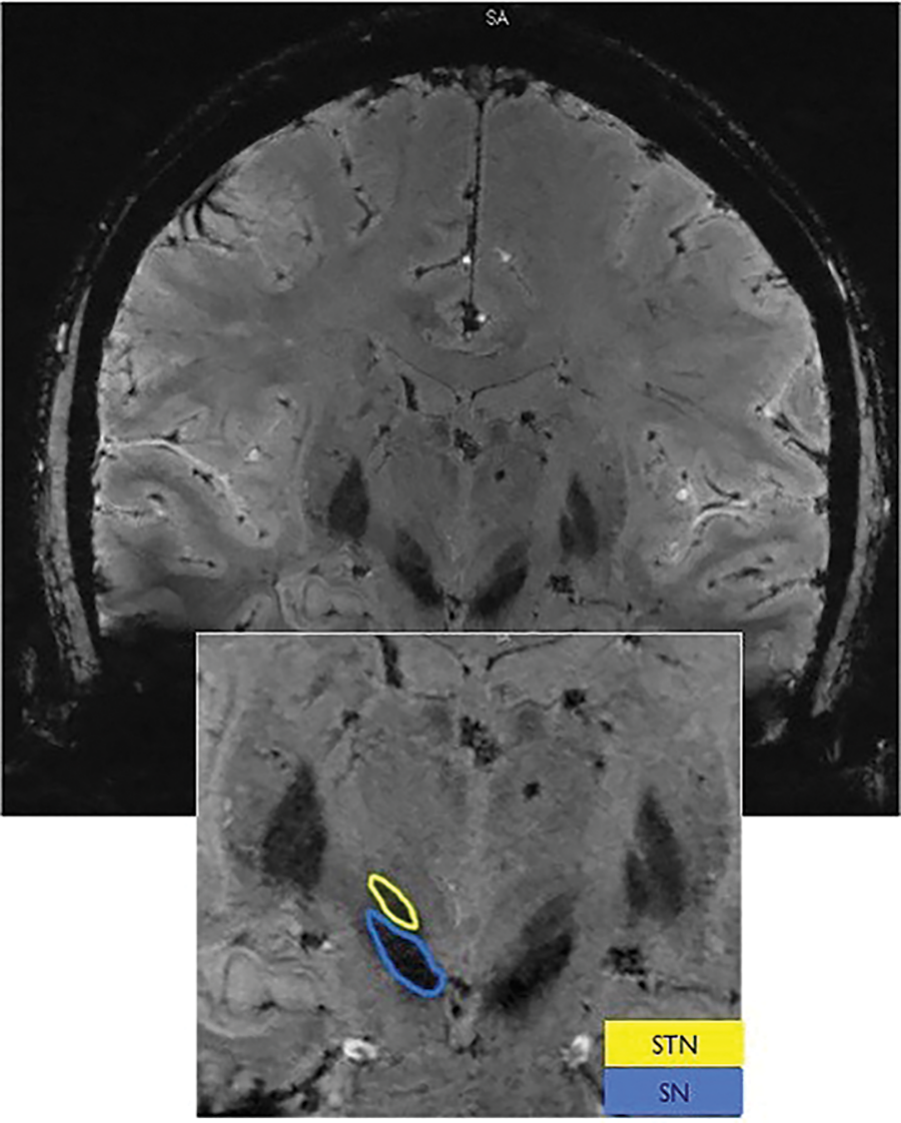
Sub-cortical anatomical structures on High-resolution 7T MR images. High-resolution 7T images are used for visualizing sub-cortical anatomical structures. As an example, the subthalamic nuclei (STN) and substantia nigra (SN) are identified on a coronal view using susceptibility-weighted imaging (SWI) contrast. Note the clear image-contrast separation between the SN and STN that allow segmentation of those structures with high confidence.

The STNs of each patient were manually segmented (voxel by voxel) on the 7T data using Amira version 5.4.1 or Avizo version 9.2 software (FEI, Hillsboro, OR, USA). The STNs were visible as a hypointense structure directly superior to the substantia nigra (SN), inferior to the zona incerta, medial to the internal capsule, and lateral to the thalamic fasciculus in the coronal plane. Fig 1 shows an example of one representative slice (SWI in this case) that was used for segmenting the STN and SN. The clear visibility of the STN borders facilitates its segmentation with high fidelity [10].

In many cases, both 7T T_2_-weighted and SWI with axial and coronal orientations were used to identify, visualize and segment these structures. Using multiple orientations and contrasts enabled us to overcome image noise and movement artifacts that are common with movement disorder patients. In order to use multiple contrast and orientation we registered the different images using affine registration (12 degree of freedom) with mutual information as the optimization metric and conjugate gradient as the optimization method. We used Amira version 5.4.1 or Avizo version 9.2 software as the registration platform. For each case, a careful inspection of the registration was performed by visually assessing the alignment of visible features of the images such as borders lines, vessels, visible nuclei, etc. We allow misalignment of up to one voxel (which can be a result of interpolation or partial volume effect). If an error larger than one voxel was detected, we improved the registration by using a different cost function (cross-correlation, mutual information), by masking parts of the volume or by changing the registration starting point. This process required several iterations of registration and evaluation. Prior to registration we resampled the images to 0.4mm isotropic using third order b-spline interpolation to preserve borders sharpness It is important to note that we did not observe any bias in the STN segmentations corresponding to the image on which the segmentation was done (T2 or SWI). This allows us to integrate the information of the segmentations from both image contrasts without having to compensate for any bias.

**Fig 2:**
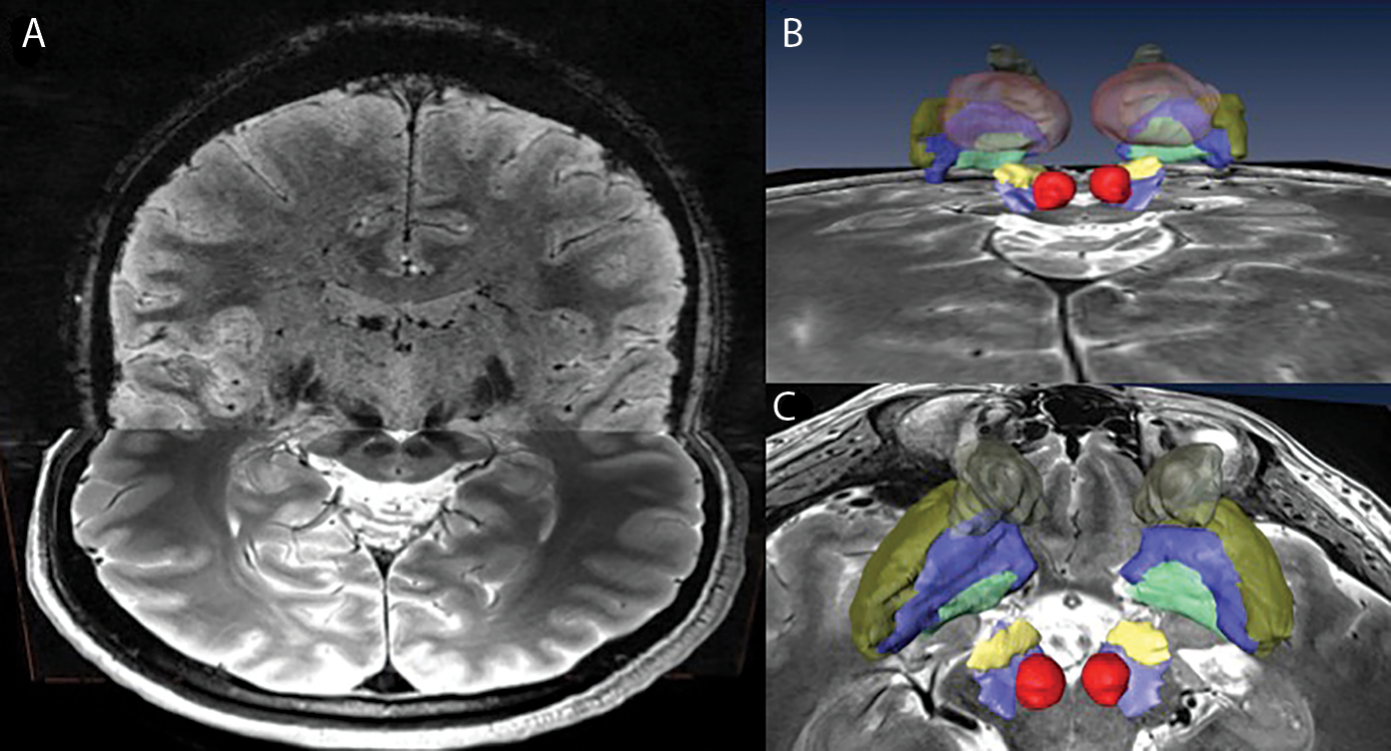
Patient-specific 3D model of multiple sub-cortical structures. Combined high-resolution and SNR 7T dataset consisting of multiple imaging contrasts and orientations (A) facilitates the creation of a full patient-specific anatomical model of the basal ganglia (B; back view) and (C; top view). The structures segmented here are: RN (red), SN (light blue), STN (yellow), GPi (green), GPe (blue), Putamen (gold) and Caudate (brown).

Fig 2 presents a patient-specific 3D model of multiple subcortical structures. This provides an example of the richness of information that can be extracted from a complete high-resolution 7T dataset consisting of multiple imaging contrasts and orientations. For this study, we chose to focus on the STN but the same technique can be used for other structures such as GPi, Thalamus, etc. The good inter-observer agreement index for segmentations of the basal ganglia based on similar images was demonstrated previously [10,11] and was further verified by independently segmented 10 STNs by two different experts and calculating their intra-class correlation (ICC). For each subject, the SN and the red nuclei (RN) were also segmented to provide a more comprehensive patient model and for ease of 3D visualization orientation. Note that while manual segmentations were used in this study, accurate semi-automatic segmentation is also possible with this high quality data [12]. We chose to use manual segmentation and not semi-automated segmentation as the accuracy of the manual segmentation is considered to be higher. A voxel was defined as STN by taking a conservative approach where only voxels for which consensus between experts was reached were labeled as STN.

### STN characteristics

**Fig 3:**
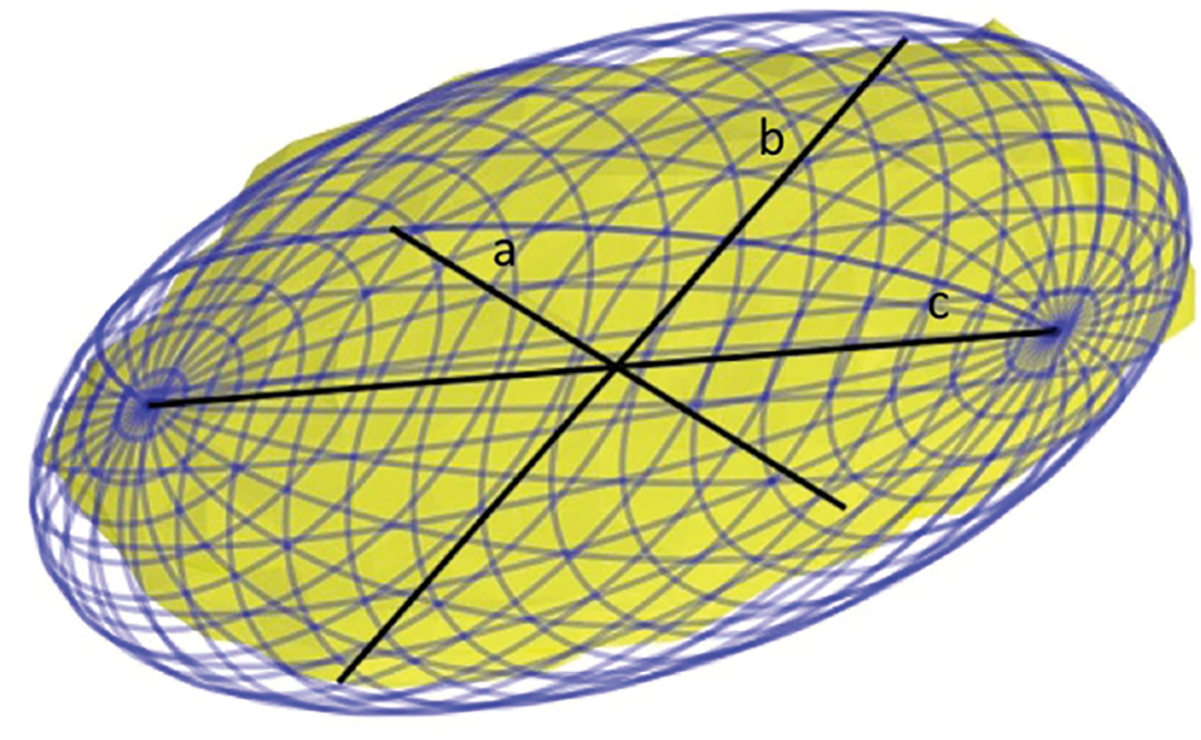
An example of minimum volume enclosing ellipsoid (MVEE) of a segmented STN volume. The MVEE (blue mesh) principal axis: length (c), depth (b) and width (a) are depicted as a solid black line. Note that a very small portion of the STN (surface in yellow) is outside of the MVEE due to the error tolerance set in the algorithm (0.001 in this case). This tolerance reduces the effect of outlier points on the final MVEE parameters.

To further understand the variability of the STN size and location between patients, we measured the following structural features of the STN:

- Volume.
- Length, width and depth as estimated by minimum volume enclosing ellipsoid (MVEE). MVEE is an approximation to the STN structure that describes its key shape parameters. The MVEE was calculated using Khachiyan Algorithm [13], that finds the MVEE of a set of data points by defining it as a minimization problem and solving it efficiently by allowing the final solution to be different from the optimal value by the pre-specified amount of tolerance. Fig 3 shows an example of MVEE (blue mesh) of a segmented STN volume (surface in yellow). The MVEE principal axes’ width (a), depth (b) and length (c), are depicted as a solid black line. Note that the length, width and depth are not necessarily aligned with the x, y and z directions (namely: medial–lateral, anterior–posterior and dorsal–ventral directions).
- Location of the STNs’ center of mass relative to the mid-commissure point (MCP). For each patient, we calculated the location of the MCP by manually localizing the anterior commissure (AC) and posterior commissure (PC) coordinates and finding the mid point on the AC-PC line. Then we transformed the image to align the y-axis with the AC-PC line and the y-z plane with the brain mid-plane that was found by selecting one more point on the mid-plane. Finally, we calculated the distances between the STNs’ center of mass and the MCP for each of the coordinates (x, y, z).

We have used python version 2.7.14 [14] as a software environment. Calculating the MVEE and the geometrical features was done using numpy 1.13.3 [15] and scipy 0.18.1 [16]. Calculating correlations, t-test, ICC, averages, standard deviations, etc., as described in the Results section, were done using statsmodels 0.8.0 [17] and pandas 0.20.3 [18]. The figures were produced using python matplotlib 2.1.0 [19].

### DBS surgery procedure

All patients subsequently underwent awake DBS surgery as part of their standard clinical care, using stereotactic CT/clinical MRI on a navigation platform (Stealth, Medtronic) with consensus coordinates used as the initial target. Consensus coordinates were generated on the imaging platform using the registered stereotactic CT/clinical MRI datasets. The target location was usually slightly modified by the neurosurgeon to meet safety and preferences requirements. The planning stage was followed by intraoperative microelectrode recording (MER) to better localize the target. The first track was aimed at the planned target while the subsequent MER tracks were aimed at verifying the target and its borders. This process is also called mapping [20]. The chronic DBS electrode was usually implanted along one of the microelectrode tracks based on two criteria: 1) The MER contained several millimeters of a typical STN neuronal activity and movement-responsive characteristics; and 2) the chronic electrode was located sufficiently medial to the lateral edge of the STN to avoid low threshold adverse effects due to stimulation of the internal capsule. Macro-stimulation was used to confirm or modify DBS electrode location based on resolution of symptoms and absence of stimulation-induced adverse effects at clinical thresholds of stimulation.

### Postoperative electrode localization

Postoperative CT imaging was performed several weeks following the day of surgery [20]. The post-surgery CT scans were obtained on a Siemens Biograph64 Sensation with the following parameters: 0.6 mm slice thickness, kV 120, 512x512 matrix, FOV 260 mm^2^, 315 mAs, with 0.0° gantry tilt. Postoperative CT scans were obtained with and without Extended Houndsfield Units (EHU), to optimize imaging of DBS contact location and minimize scattering artifact from the DBS electrode while offering good brain contrast and electrode localization. Performing the post-operative CT scan several weeks after the surgery allows enough time for the brain tissue to recover from “brain shift” that might occur during surgery. The presence of brain shift would likely reduce the accuracy of preoperative-to-postoperative image registration and the localization of the DBS electrode on postoperative image [20].

The electrode 3D model was semi-automatically extracted using either Amira version 5.4.1 or Avizo version 9.2 (FEI, Hillsboro, OR, USA). Then, an exact 3D representation of the specific electrode implanted in the patient was matched with the segmentation. The 3D electrode model was generated following the dimensions provided by the vendor and included a shaft, a tip, and four contacts (in all cases the electrode model was Medtronic DBS lead 3389). The pre-operative 7T MRI and post-operative CT scan were linearly co-registered using Amira version 5.4.1 or Avizo version 9.2 software. Since it is hard to register a thin MRI slab to whole brain CT, we usually used the T1 as an intermediate registration target. Once we achieved an adequate registration between the 7T MRI T2 or SWI and the T1, we registered the T1 to the CT and concatenated the transformations. These registrations were achieved using an affine registration. The cost function chosen was Mutual Information while the optimization method was conjugated gradient. If these parameters did not yield a satisfactory registration, other cost functions, registration starting points, registration masks and optimization methods were explored until the registration was deemed good. The registration was verified visually by testing the alignment of visible features such as ventricles, sulci, blood vessels, nuclei in the Basal Ganglia, etc. Emphasis was given to the accuracy of the registration of the mid-brain region. This allowed for better registration in the region of interest at the expense of lower registration accuracy at the peripheral area that is less relevant for STN-DBS. This process facilitated the visualization of patient-specific anatomical 3D structures along with the electrode model in the same space. Finally, the active contact was determined based on a monopolar survey conducted by a health care professional at the time of initial programming (typically one month after the surgery). In the monopolar review, the caregiver systematically stimulates the STN with various DBS settings to find a setting that maximizes the patient benefits while avoiding adverse effects.

### MER validation

To validate the accuracy of the imaging-based model with the current gold-standard, which is the neurophysiological activity as measured by MER, we compared the lengths of the electrode trajectory within the STN as measured with MER along the implanted electrode trajectory, with the patient-specific 3D anatomical model based on 7T MRI. The MER STN entry and exit points were inferred based on the intraoperative notes that were taken during the surgery.

### Patient-specific STN—VTA analysis

To demonstrate that the accuracy of the patient’s own STN shape and location is relevant for clinical outcomes, we computed the correlation between the patients’ motor improvement and the STN overlap with the volume of tissue activated (VTA) in 12 subjects for which monopolar reviews were available. Monopolar review charts were used to extract the patient’s motor improvement and their associated stimulation parameters. All patients were off-medication at the time of the monopolar review. At each session, motor function of the opposite side to stimulation was evaluated. Assessment of motor function typically included rest tremor, finger taps and hand movements. Other measures were incorporated when the caregiver found it necessary. As commonly performed in standard clinical care, a single caregiver, either a neurologist or a nurse practitioner, assessed the patient’s motor function off-stimulation and off-medication before testing each of the contacts. Then, the amplitude of the stimulation was increased in steps of 0.5V and the motor function of the patient was evaluated. Note that we use the monopolar review as a proxy to the patient outcome since we did not have access to the patients’ full UPDRS-III evaluation for each stimulation setup. Typically, 20–30 DBS setups are tested during the postoperative follow-up monopolar review visit that can take several hours. It would be impossible to perform a complete UPDRS III for each tested setup since it would require a very long time. Instead, the examiner usually selects 2–4 prominent symptoms, most often contralateral upper limb's tremor, rigidity and bradykinesia, and estimates their improvement with DBS stimulation, off medication. The most common tests were rest tremor, finger taps and hands rotation. The scoring method was done according to the method described in the UPDRS-III instructions that were published by the Movements Disorders Society [21]. The examiner scored each symptom severity between 0 (no symptom) to 4 (severe symptom appearance). For example, the instructions given to the examiner for the finger tapping grading were as follows:

Each hand is tested separately. Demonstrate the task, but do not continue to perform the task while the patient is being tested. Instruct the patient to tap the index finger on the thumb 10 times as quickly AND as big as possible. Rate each side separately, evaluating speed, amplitude, hesitations, halts and decrementing amplitude.

0: Normal performance: No problems were observed.
1: Slight difficulty performing the task characterized by: any of the following: a) the regular rhythm is broken with one or two interruptions or hesitations of the tapping movement; b) slight slowing; c) the amplitude decrements near the end of the 10 taps.
2: Mild difficulty performing the task characterized by any of the following: a) 3 to 5 interruptions during tapping; b) mild slowing; c) the amplitude decrements midway in the 10-tap sequence.
3: Moderate difficulty performing the task characterized by any of the following: a) more than 5 interruptions during tapping or at least one longer arrest (freeze) in ongoing movement; b) moderate slowing; c) the amplitude decreases after the 1st tap.
4: Severe difficulty performing the task: the patient cannot or can only barely perform the task due to lower tapping frequency, interruptions in the tapping sequence or decrease in the amplitude of the finger tapping.

We averaged the scores for the selected tasks and normalized the result by dividing the average score with the average score off-DBS off medication. Then, the motor improvement was converted to percentage.

For each stimulation parameter assessed (specific values of contact number, amplitude, and resistance) we computed the VTA using the model suggested by Madler et al. [22]. In this study, only monopolar settings were used. This multivariate polynomial fitting method creates an ellipsoid around the stimulation contact of interest for which the size is dependent on the stimulation voltage and electrode impedance. A binary image that represents the VTA was then generated and we computed the overlap of the VTA with the STN that was segmented on the 7T MR image. Finally, we computed the percentage of the STN that was covered by the specific VTA. The correlations between the motor improvements and the overlap of VTA and STN were then computed with Pearson’s correlation coefficients.

The VTA calculation was done using Python numpy 1.13.3 and geometrical operation such as calculating structures’ surfaces and overlaps was done using VTK 7.0.0 [23] python module.

## Results

### STN structure–variability analysis

**Fig 4:**
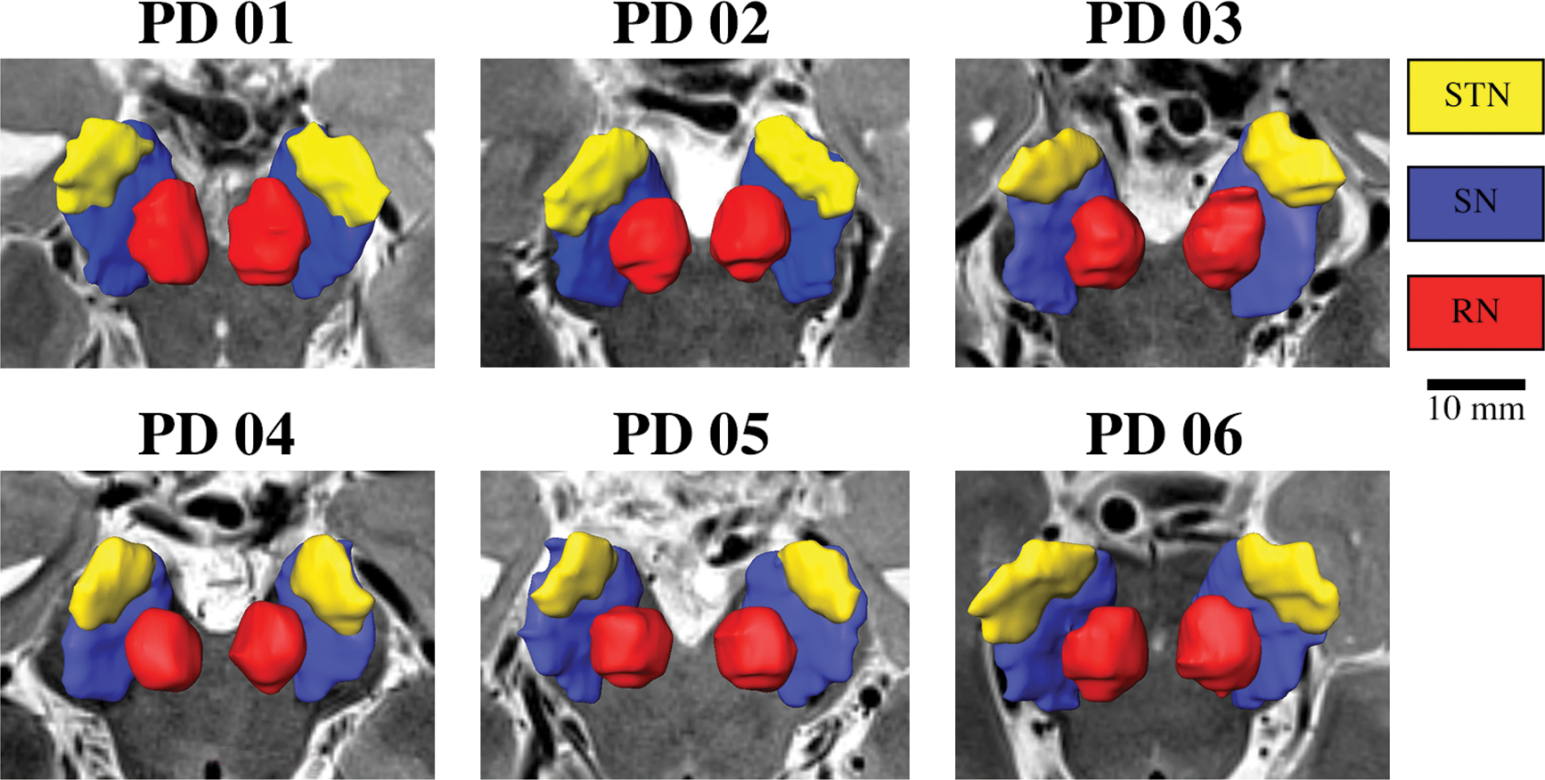
Example of anatomical 3D reconstruction of the STN, SN and Red nuclei. Anatomical 3D reconstruction of the STN, SN and Red nuclei of six different patients imaged with 7T MRI. Note the variability in the shape, size and location of the structures across individuals.

Great variability in the size, shape and location of the STN was observed (see Fig 4 for a qualitative depiction of six patients.)

**Fig 5:**
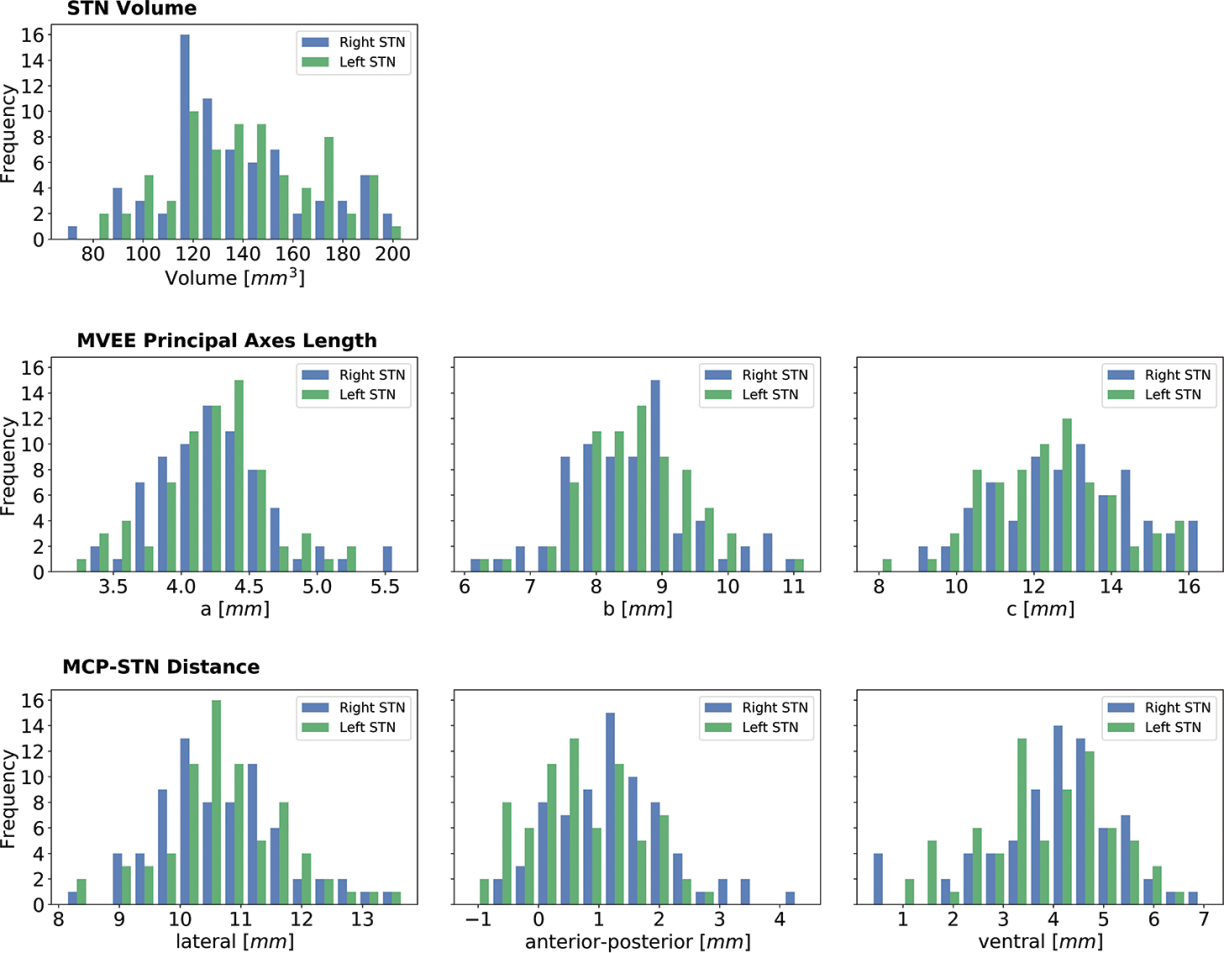
Variability analysis of the STN structure (N = 144). The first row shows the volume distribution of the STN. The second row depicts the distribution of the STN dimensions as estimated by MVEE principal axes. The third row depicts the distribution of the STN center of mass location relative to MCP. The results are in agreement with previous findings [1,24]. Note that previous studies measured the STN dimensions visually on MRI axial plan (extrinsic image coordinates). However, the MVEE principal axes (a, b, c) capture the intrinsic STN dimensions across multiple patients independent in the image orientation. The principal axes are not necessarily aligned with the x, y, z directions and therefore the length reported here is longer than that reported previously.

**Fig 6:**
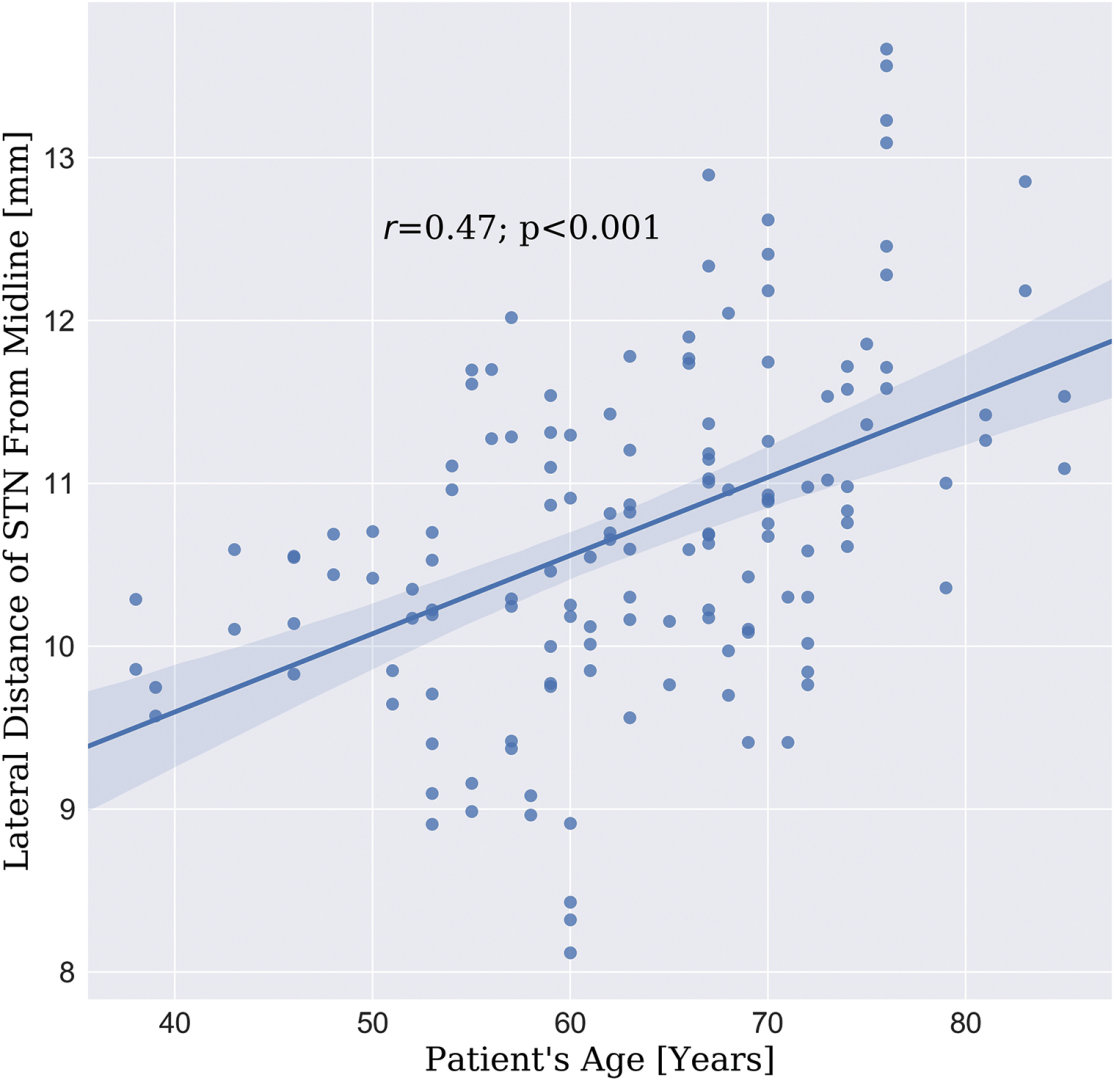
STN lateral location correlation with patient age. The correlation (r = 0.47; p < 0.001) between the patient’s age and the lateral distance of the STN center of mass from the midline.

To quantify this variability, we measured the following parameters across 72 subjects (n = 144 STNs) and the results are as follows (average ± SD):

1. STN volume was 137.1 ± 29.4 mm^3^ and 140.4 ± 29.0 mm^3^ for right and left STN, respectively. No significant difference was found between the right and left STNs in all categories (t-test p > 0.2 Bonferroni corrected)
2. Based on the minimum volume enclosing ellipsoid (MVEE) the characteristic lengths of the main axes of the STN were measured to be (length, depth and width) 12.3 ± 1.7 mm, 8.5 ± 0.8 mm and 4.2 ± 0.4 mm for the right STN. For the left STN, these lengths were 12.9 ± 1.8 mm, 8.5 ± 1.0 mm and 4.3 ± 0.4 mm. Note that the shape-intrinsic length, width and depth are not necessarily aligned with x, y, z directions (namely: medial–lateral, anterior–posterior and dorsal–ventral directions) and therefore, the dimensions of the STN measured here can be slightly different than the conventional way of measuring the STN along the extrinsic image coordinates. Principal axes are a better way to capture the structure’s dimension for systematically comparing the intrinsic STN dimensions across multiple patients without dependency in the image orientation.
3. The distance from the STN’s center of mass relative to the mid-commissure point (MCP) was measured to be (left-right, anterior-posterior and inferior-superior axes) 10.8 ± 1.0 mm, 0.7 ± 0.9 mm and 3.8 ± 1.3 mm for the right STN. For the left STN, these distances were 10.7 ± 1.1 mm, 0.9 ± 1.0 mm and 4.0 ± 1.3 mm. A summary of the quantitative measure is depicted in Fig 5. The figure presents the variability in the location of the STN relative to MCP, the shape parameter (MVEE) and the volume, respectively.
4. We observed a significant Pearson’s correlation coefficient (r = 0.47; p < 0.001) between the STN’s center of mass distance from the midline (lateral distance) and patient’s age (see Fig 6).
5. We did not find any significant difference (t-test p > 0.2) between the STN size and location for ET and PD cohort.

### Final electrode location

**Fig 7:**
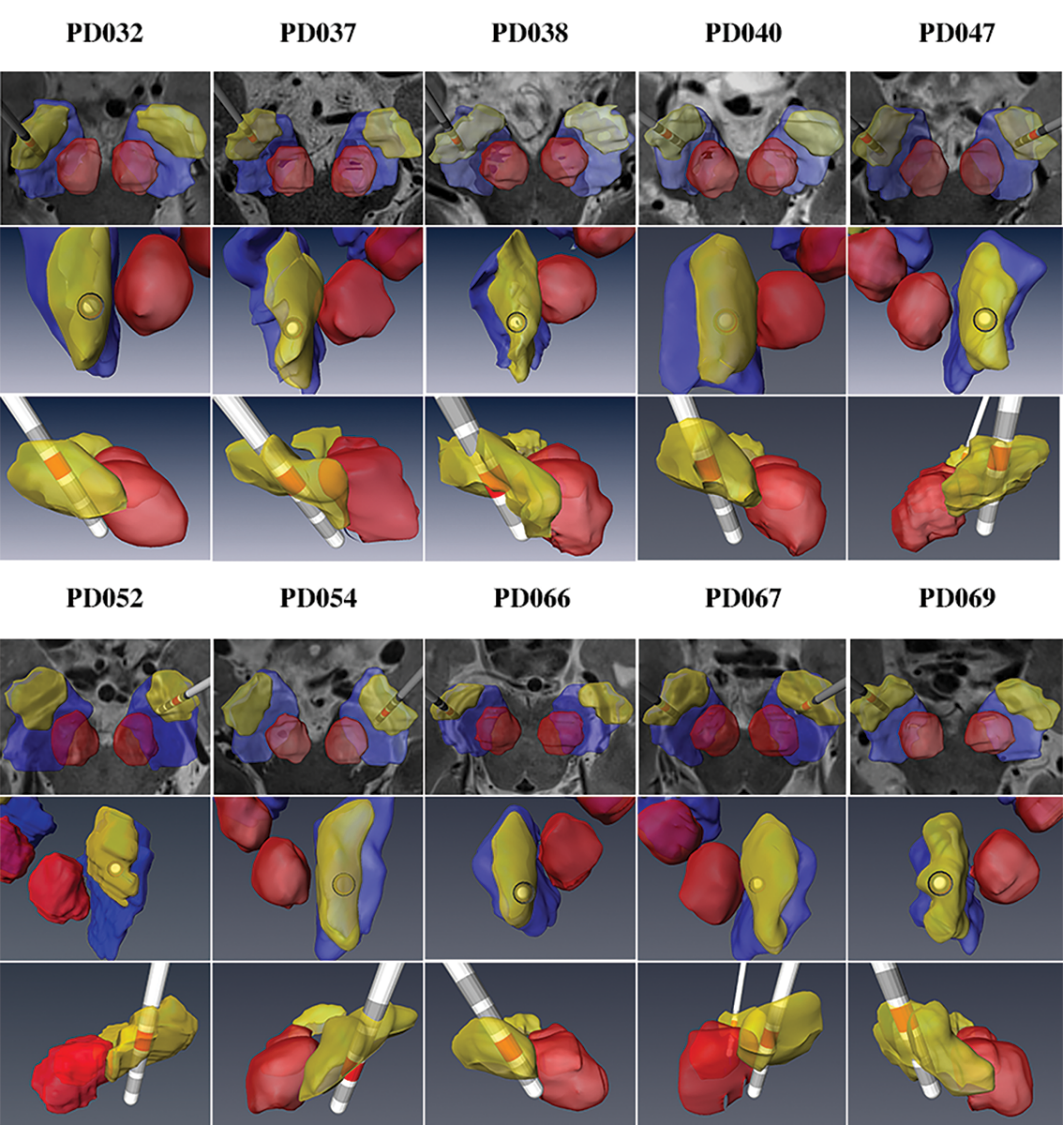
3D models of the STN and the implanted electrode. Patient-specific anatomical 3D models of the STN and the implanted electrode for ten Parkinson’s disease (PD) patients are shown. We present three different view angles to better visualize the DBS electrode location in 3D space. The active contact, as defined by the monopolar survey, is marked in red.

For all the patients implanted in the STN, the reconstruction of the electrode and its contacts revealed that the final location of the electrode was indeed in the posterior half of the STN (see Fig 7 for a 3-dimensional representation of the patient-specific post-operative model across 10 patients).

### MER validation

**Fig 8:**
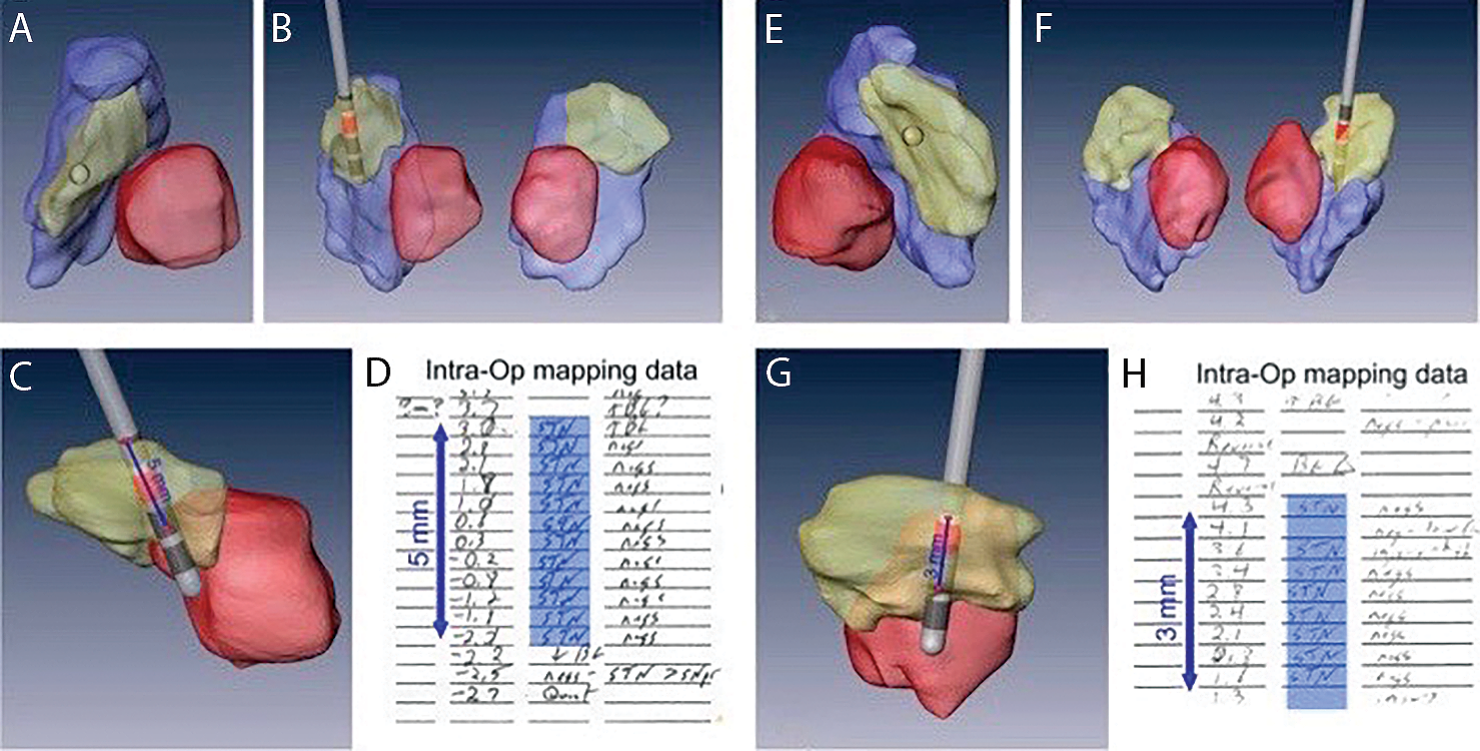
Example of MER notes versus the implanted electrode trajectory. Examples of two Parkinson’s disease (PD) patient-specific anatomical models of subcortical structures along with implanted electrode and active contact (red). A and E provides axial view. B and F coronal view, and C and G sagittal view with the implanted electrode. D and H are the MER notes taken during the surgery of the final tract where the DBS lead was implanted. Excellent agreement between the MER data and the model was found.

**Fig 9:**
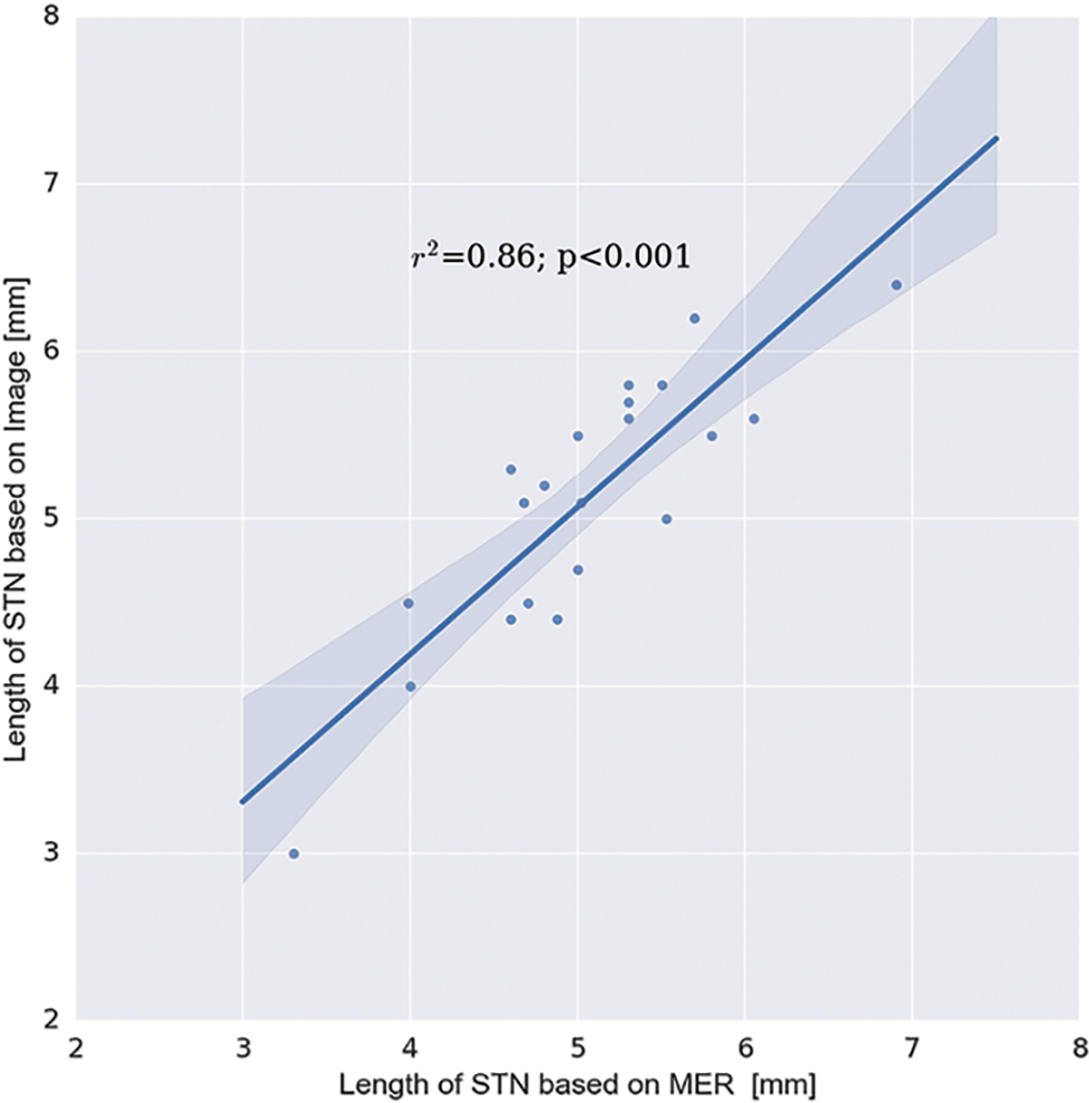
Correlation between STN trajectory lengths based on MER vs. 7T model. Correlation between the length of the STN, as defined by MER at the DBS lead location, and the length of the STN as defined by the 7T images. The data indicates excellent agreement between the 3D 7T imaging-based anatomical model of STN and the corresponding MER mapping. The shadow represents the 95% confidence interval around the regression line.

We have obtained the intraoperative MER notes of the track that was used for implanting the DBS lead for 21 cases. For the remaining cases, we were unable to get detailed MER notes. Using the MER notes to determine the actual length of the STN traversed (based on the location of the entry and exit points), we observed an excellent agreement between the imaging-based 3D anatomical model of the STN and the MER mapping (see Fig 8 for an example of patient-specific anatomical models for two PD patients). In fact, we observed a significant Pearson’s correlation coefficient (r = 0.86; p < 0.001) and Lin’s concordance correlation coefficient [25] (ρ = 0.86; p < 0.001) between the measures (see Fig 9 for a scatterplot comparing the lengths of the STN traversed by the electrode based on MER and our 3D anatomical model).

### VTA analysis

**Fig 10:**
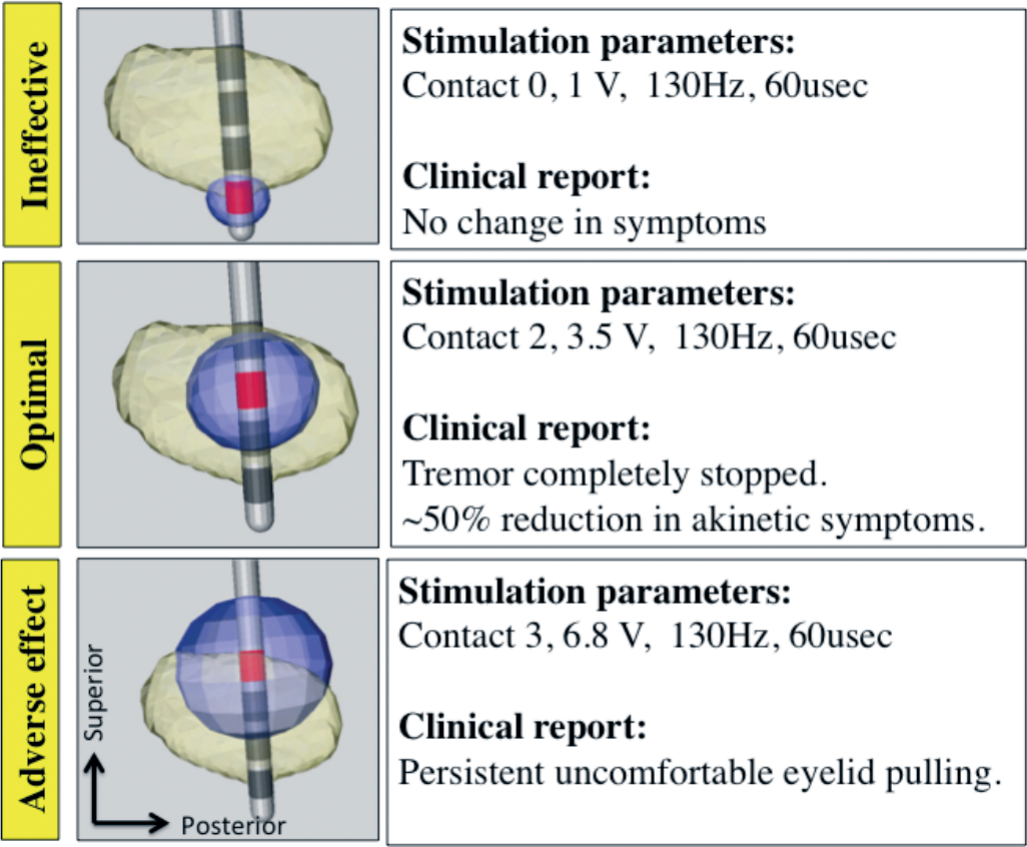
Example of VTA-STN overlap. An example of the computed VTA for three different stimulation settings relative to a representative case of patient-specific STN. For each stimulation setting, the clinical report, as written in the monopolar review, is presented. It can be seen that minimal overlap with the STN resulted in no benefit, good overlap resulted in optimal benefit, and good overlap while stimulating large volume outside the STN resulted in a significant adverse effect.

**Fig 11:**
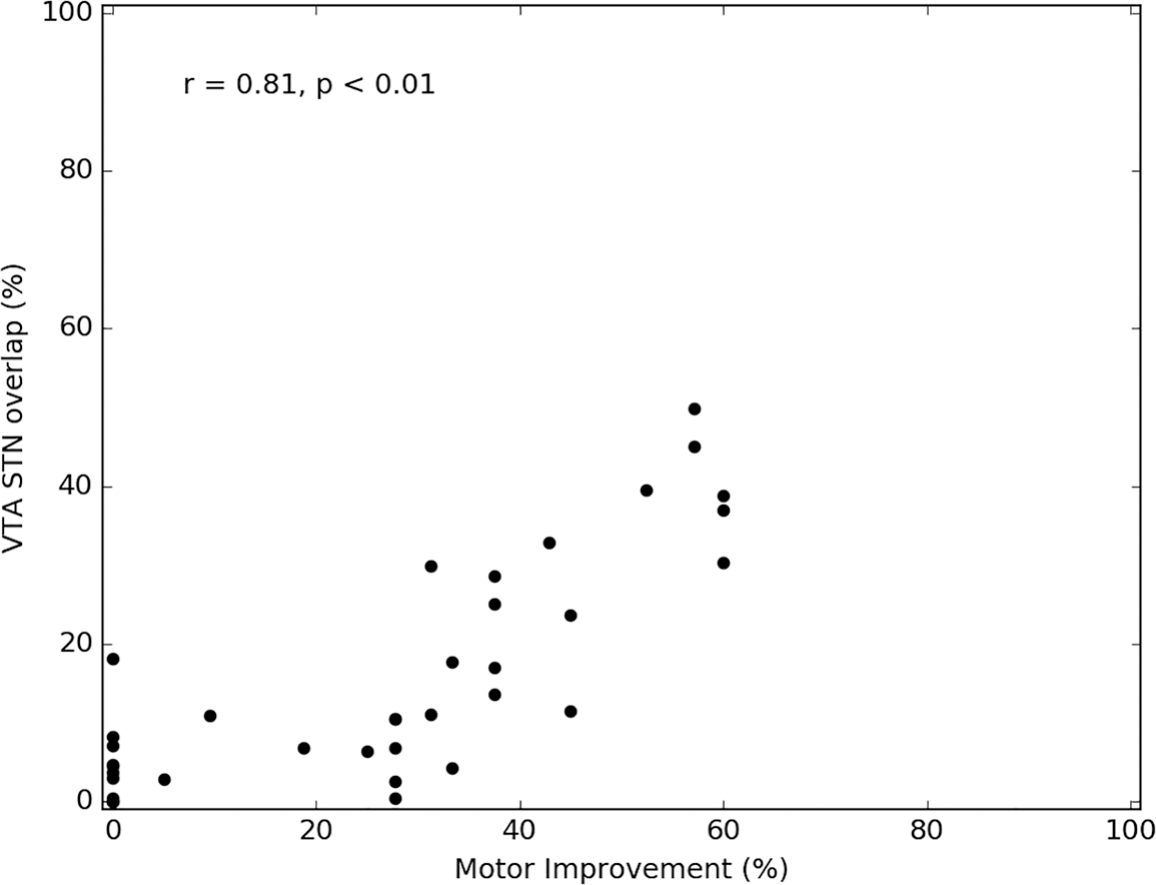
Correlation between VTA–STN overlap and motor improvement. An example of a correlation between the VTA overlap with the STN and a patient’s motor improvement scores as were evaluated during a monopolar review session (see methods section for details). The Pearson’s correlation coefficient in this case was r = 0.81 (p < 0.001).

All patients have demonstrated significant (p < 0.05) Pearson’s correlation coefficient between motor improvement and the VTA of the STN for each setting tested during monopolar review (0.61 ± 0.16 (average ± SD); range 0.38–0.86). Fig 10 depicts the computed VTA for three different stimulation settings relative to a representative case of patient-specific STN. For each stimulation setting, the clinical report, as written in the monopolar review, is presented. The patient experienced clinical benefit when the VTA covered a substantial portion of the posterior section of the STN that incorporates the motor territory as was shown by Plantinga et. al. [11]. However, when the VTA also incorporated a significant volume outside the STN, the patient experienced adverse effects. Fig 11 shows the correlation between the VTA overlap with the STN of a given patient and the patient’s improvement score (see methods section for details). The correlation in this case was r = 0.81 (p < 0.01).

## Discussion

Exact localization of the anatomical target structure and the precise placement of the electrode within it are critical factors in the success and effectiveness of DBS treatment [2,26]. While the DBS target is functional and not anatomical, the functional target can usually be associated with the anatomical definition [11]. Yet, the exact optimal target for STN-DBS is still under debate [27]. The current practice of placing a DBS electrode incorporates indirect and direct targeting approaches. Indirect targeting provides some form of consensuses coordinates based on an atlas-based target. In order to improve the accuracy of DBS electrode placement, the direct approach requires the neurosurgeon to adjust their planning based on a clinical MRI T2 or FLAIR axial view where the STN appears as a hypointense area while others may also incorporate the location of subcortical structures landmarks such as the red nucleus [28,29]. However, the image resolution and the low signal-to-noise-ratio usually does not allow for clear identification of the target or its borders. For example, Verhagen et al. [30] observed significant difference between the anatomical STN borders (lateral and dorsal) as defined by 1.5T and 3T MRI and the borders as defined by neurophysiology as measured by MER. As shown here, the variability of the location, shape and size of the STN is significant between individual patients (see Fig 5). Note that the standard deviation of the STNs’ center of mass is the same order of magnitude as the characteristic dimensions of the STN. Therefore, the ranges of the measured STN volume (70-200mm^3^), size (i.e., length of 10-16mm) and location (i.e., 8-13mm from midline) suggests that a one-size-fits-all atlas cannot provide reliable localization of the STN and consequently reliable targeting of the STN [5]. Furthermore, failing to accurately localize the STN may explain, in part, the inconsistency of clinical outcomes [2,3,26] in DBS surgeries. It should be noted that we report the STN center only as a statistical measure of the STN location to demonstrate its high variability. The optimal therapeutic target within the STN area is still under debate [27].

The significant Pearson’s correlation coefficient (r = 0.47; p < 0.001, see Fig 6) that was observed between the STN’s center of mass distance from the midline (lateral distance) and the patient’s age is in agreement with Keuken et al. [31] and Pereira et al. [32]. The STN average volumes measured in this study (140.4 ± 29.0 mm^3^; 137.1 ± 29.4 mm^3^ for left and right STN, respectively) are in agreement with other studies that estimated the STN size [10,33,34].

This work aims to facilitate the approach of direct targeting for functional neurosurgery that overcomes the high variance in STN localization. Capitalizing on the advantages of ultra-high field MRI (7T) system, a patient-specific anatomical model is created, depicting the patient’s own anatomical features (Figs 1 and 2). Relying on the MER as the “gold standard” for localizing the DBS structure of interest (STN in this case) enabled us to validate the accuracy of this patient-specific imaging-based model. Excellent agreement was found between the anatomical structures as defined by the imaging and the MER notes that define the anatomical structures based on their functional properties (Fig 9). The correlation (0.86) and Lin’s concordance coefficient (0.86) suggest that the STN as defined functionally by the MER and anatomically by the 7T MRI are of equivalent shape and position (see Fig 8 for example). Note that in this study the entry and exit points from the STN, observed using MER, were determined by the neurophysiologist based on subjective estimate of the point where the STN neural activity is above the baseline noise. Previous studies reported that the STN extends further in the dorsal direction than is measured by MER [30]. However, we did not find such a bias in this study.

Another benefit of the presented method is that it provides an accurate 3D model of the patient-specific STN, in vivo and non-invasively, in contrast to the accurate but one-dimensional MER or the 3D but inaccurate atlas-based models. Such a model, directly derived from 7T MRI data, can support the clinical decisions for effective DBS. For example, analyzing Fig 8 the patient on the left (A-D) shows a 5mm path through the STN, while the patient on the right (E-H), where the lead was located slightly more anterior-medial, the MER shows only 3mm of STN neural activity, as the anatomical model predicts. Analysis of the model suggests that using a different entry angle for this patient may have provided a longer path through the STN, manifesting the value of patient-specific anatomical models for planning and programming of DBS surgeries. A longer path through the motor part of the STN is considered better since it usually allows for a wider therapeutic window and a better chance to find stimulation settings that alleviate patient symptoms while avoiding adverse affects. Zaidel et al. observed that the spatial extent of the dorsolateral oscillatory region in the STN correlates with the outcome of STN-DBS [35]. In addition to enabling better planning for approaching the intended target (i.e., optimization of the entry point and the entry angle), better direct targeting could minimize the number of MER passes required to localize the target, hence, increasing the safety, shortening surgery time and allowing better outcomes of the procedure. The excellent agreement between the MER and the 7T MR imaging suggests that 7T MR images of the basal ganglia may be used as the new gold standard for localizing these structures. Such an accurate 3D model may provide the rationale for developing additional approaches to target those structures or to better understand morphological changes in those structures that are related to different disease stages.

Following surgery, the 3D model can accurately estimate the implanted electrode location with respect to different sub-regions of the STN [11] (as shown in Fig 7). The electrode location can be computed from the postoperative CT. This enables one to analyze the relationship between DBS electrode location relative to the STN and therapeutic outcomes at a higher level of detail with much greater accuracy in comparison to atlas-based method. This will, in turn, enable a much higher level of accuracy in determining the optimal settings.

The high correlation values that were observed between motor improvement and the overlap of STN with VTA (Fig 11) further validates the accuracy of the patient-specific STN model and demonstrates the clinical relevancy of our method. Note that the correlation values observed here are better than those reported in other studies that incorporate an atlas to define the STN [36,37]. It should be noted that current commercial and research systems to support clinical decisions regarding the stimulation parameters use a standard atlas of the basal ganglia [38]. Therefore, our results suggest that the correct placement of electrode within the actual STN will be associated with greater motor improvements. Moreover, the accurate definition of STN boundaries may be a key factor for safe and consistently effective DBS treatment. Given that VTAs outside of the STN in one direction may be associated with adverse effects, while potentially beneficial in other directions–accurate placement of the lead and postoperative reconstructions of lead location for optimizing stimulation settings further emphasizes the importance of this approach for maximizing clinical outcomes. It should be noted that accurately localizing the STN borders might prove to be crucial for programming guidance for the newly directional leads since it allows further optimization of the exact region within the STN to be stimulated. The high correlations we observed suggest that using the 7T patient-specific 3D model may facilitate a more informed and confident clinical decision-making. Furthermore, combining the patient-specific 3D model of the STN and nearby structures with an optimized VTA may open the door to a better understanding of DBS mechanisms (e.g. whether there are specific regions within and/or around the STN that are specific for particular motor signs) and provide the rationale upon which we can determine the optimal electrode location for each patient that will maximize benefits.

**Fig 12:**
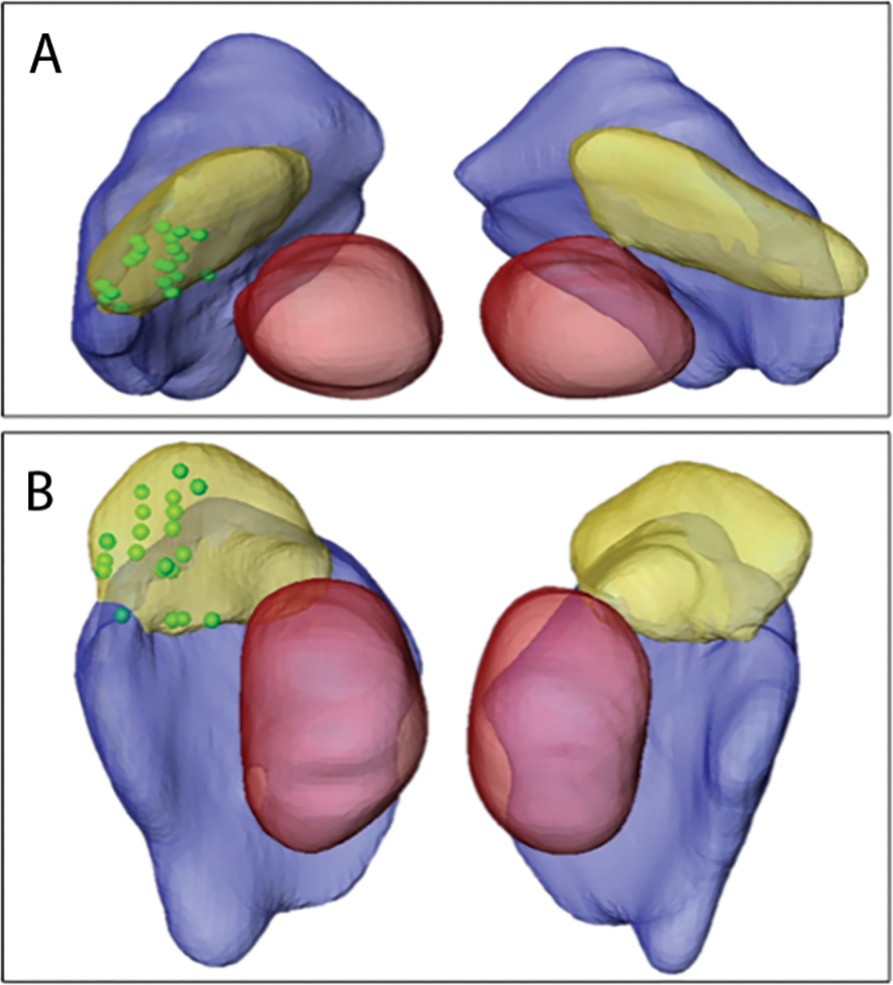
Active contacts on averaged STN. The locations of the active contacts are superimposed on an average STN model in axial (A) and coronal (B) view. Green spheres: location of the active contact for different patients.

DBS outcomes vary considerably among PD patients, and lead misplacements can be associated with adverse effects [3]. We expect that patient-specific targeting will assist in minimizing such adverse effect and maximize treatment efficacy. An example of such an approach is presented in Fig 12 where the locations of the active contacts, as determined in the monopolar review, of N = 20 patients were superimposed on a representative 3D STN model. The locations of the active contacts (presented as green spheres) on the representative 3D STN model were determined by considering the location relative to the patient’s own STN borders and center of mass. This cluster of points enables one to correlate the location of the active contact and its VTA to clinical outcome. Note that the contacts locations were determined solely by the neurosurgeon and the neurophysiologist based on their preferences without the use of our 3D model. The variability in the active contact location is apparent although all of them appear to be located in or in adjacent to the motor part of the STN [11]. A more comprehensive and quantitative study on using a 7T MRI-based patient-specific STN segmentation to correlate the VTA with patient outcome and calculate optimal stimulation settings is currently under preparation.

The improvement in targeting by using 7T imaging may facilitate asleep DBS, alleviate some of the anxiety many patients experience with awake procedures. [39].

Finally, we are aware that the availability of 7T MRI for clinical use is limited although the latest Siemens Magnetom Terra 7.0T scanner received FDA and CE approval for clinical use. Nevertheless, the ability to construct an accurate 3D patient-specific STN model may pave the way to develop novel computational techniques to detect the STN location even on lower quality images [40].

### Caveats, pitfalls and mitigations

While patient-specific 3D STN models based on 7T MRI appear to be accurate and agree with the validation methods we employed, there are still inherent sources of errors and inaccuracies in this model. The following section discusses these various sources and the mitigation used to minimize them.

#### Image and contrast resolution

The upper limit of the accuracy of the 3D imaging-based model is as good as the resolution of the input images. In our case, the resolution of 0.4x0.4mm in-plane and 0.8mm or 1mm slice thickness for SWI and T2 contrasts, respectively, set an upper limit to the accuracy of the STN 3D model. Since the STN is a small structure relative to the image resolution, about one fourth of the voxels representing it are along its surface, where segmentation uncertainty is higher due to partial volume effects [41]. In addition, motion artifacts, common when scanning PD patients, may introduce further blurring of the image, making the task of accurately segmenting small structures very challenging. Note that in small structures such as the STN where the characteristic width is about 4mm, a single voxel of 1mm accounts for 25% of the structure’s width. Using anisotropic voxel size may also affect the morphometric measures of subcortical structures as shown by Wonderlick et al. [42]

We were able to partly mitigate these issues by using multiple contrasts (T2-weighted and SWI) and orientations (coronal and axial) hence allowing for better resolution and differentiation around the border area and compensating for the anisotropic voxel size. Both T2-weighted and SWI images were acquired in order to view the structures and their surroundings using different contrast and, therefore, increase our confidence in the segmentation. For example, SWI is a 3D acquisition where the contrast-to-noise ratio of the STN is very high. However, this type of acquisition is very sensitive to motion of the patient during the scan (as it sometimes happen with motor disorder patients). In those cases, we use the T2-weighted MRI, which is a 2D acquisition and less sensitive to motion artifacts. Note that we do not claim that our segmentation method is superior to other segmentation methods. We simply describe a process that allows us to segment a small structure in a way that increased our confidence in the resulted segmentation. We should emphasize that it is a common practice for the neurosurgeon to plan the trajectory with the longest path through the STN with movement-responsive cells while avoiding placing a potential active contact at the borders of the target to minimize side effects that may result from stimulating adjacent structures (i.e. the internal capsule). We therefore suggest that while it would be beneficial to have structures’ borders accurately segmented, an accuracy of 1mm around the borders is sufficient to enable effective and safe STN-DBS. In the case where an application requires higher accuracy, image acquisition with higher resolution will fulfill such requirements as model accuracy and image resolution are dependent on one another.

#### Manual segmentation process

The segmentation process was done manually (voxel by voxel) and therefore we do not expect any biases resulting from using a specific software for segmentation. However, there can be segmentation biases due to human conventions. For example, our group chose to be conservative in the segmentation process and only label voxels as STN voxels when a consensus between raters could be reached. The borders of the STN are sometimes not clear even on a 7T MRI images, this could lead to a bias that underestimates the STN size. Nevertheless, no such bias was found in the correlation between the STN segmentation and the functional MER records.

To measure the inter-rater reliability we independently segmented 10 STNs by two different experts. We then used intra-class correlation (ICC) as our measure for inter-rater and intra-rater reliability. We also segmented again 10 STNs about three months after the first segmentation by the same expert in order to measure intra-rater reliability. The inter-rater and intra-rater reliability were measured to be 0.85 ± 0.09 and 0.90 ± 0.07 respectively.

#### Guidance system

Another source of error might arise from an inherent error of the stereotactic system software (in our case Medtronic Stealth FrameLink) and the tolerances of the hardware (in our case Leksell Stereotactic Head-frame [43]). The error of the stereotactic system usually stems from two sources: a) the accuracy of the registration, and b) the defined mechanical tolerances of the head frame. The error attributed to these sources is estimated by the vendor to be around 1-2mm [43,44]. Note that these errors only influence the accuracy of intra-operative targeting and not the post-operative analysis since in the latter we are only interested in the relative location of the DBS target and the DBS electrode. The error in that case is minimal due to the use of multiple orientations to verify the quality of the registration between the pre-operative and the post-operative images and is estimated to be around one voxel size, which is around 0.5mm.

#### Geometrical distortion

A common concern regarding high field MRI is that it is prone to larger geometrical distortions than standard clinical scanners, which may reduce the ability to use its images in clinical operations. In previous work [8] it has been shown that while distortions do exist in 7T images, they can be minimized by using carefully optimized MRI acquisition protocols. In that case, the distortions are comparable to those found in a clinical MRI, routinely used for guiding surgical operations. This is especially true in the mid-brain where the DBS targets are located. A recent work by Lau et al.[45] further supports the notion that distortions in the midbrain region are minimal and therefore it is possible to achieve adequate registration in the mid brain sometimes at the expense of less accurate registration in the peripheral brain regions that are more susceptible to distortions. Note that usually there are still small distortions even after the MRI scanner manufacturer’s own distortion correction (Siemens). These distortions differ for different acquisition orientations and contrasts. Since we insist on a minimal registration error (up to one voxel) we used affine registration, which we found to correct most of these small distortions. The amount of scaling and shearing in the transformation matrix are usually very small (up to 3% scaling and close to zero amount of shearing). Furthermore, affine registration of the MR volumes to a CT volume, that is less prone to geometrical distortions, usually minimizes these distortions assuming that the local distortions are small and that the leading term of the distortion is linear (at least in the mid-brain area). The high correlation between the MER measurement and the clinical outcome provides evidence that the distortion of the MRI, if any, was corrected by registering to the postoperative CT.

#### Brain shift

One of the pitfalls often pointed out regarding image guided targeting is that brain shift, which often occurs during the DBS surgery may reduce the accuracy of lead placement [44,46]. The brain shift increases with surgery time, especially for patients with large ventricles and/or large brain atrophy, and could be up to few millimeters in deep structures. This caveat may be overcome by using patient-specific models for targeting since the implementation of these would facilitate hitting the target and, in turn, can decrease the number of MER tracks required for localizing the target, hence, reducing the time of operation and minimizing brain shift [47,48]. Furthermore, incorporating intra-operative CT or MRI allows for continuously updating the location of the target on the image by registering the preoperative MRI where the DBS target is visible with the intra-operative scan. Again, this may only influence the intra-operative targeting. The post-operative analysis is minimally affected by brain shift since the postoperative CT was taken several weeks after the operation where the brain returned to its normal position and no shift was observed.

## Conclusion

STN segmentation based on 7T MRI is shown to correlate well with the STN boundaries as determined using MER. Considering that most centers do MER mapping in only one plane, and therefore provides only 1D definition of the STN, there is a great benefit in using a patient-specific 3D STN model to target the STN. Moreover, the high correlations with the monopolar review outcome demonstrate the clinical relevancy of such patient-specific 3D model and further validate its accuracy. Our findings of the variability in STN location and dimension suggest that patient-specific 3D STN models are preferable to any brain atlas. We are aware that 7T MRI machines are currently not available for the majority of clinical centers. However, using 7T MRI as the ground truth for detecting the STN can be used to estimate the accuracy of other methods to target the STN and pave the way for improvement of these methods.

Better accuracy in identifying the DBS targets facilitates better surgical targeting, and can potentially reduce the number of MER penetrations, shortening the time of surgery, and reducing the variability of surgery outcomes [2,26].

In summary, high field (7T) MRI provides a unique opportunity to create an accurate and reliable patient-specific 3D anatomical model of the basal ganglia DBS targets and their adjacent structures. Such accurate models may facilitate a range of applications such as DBS surgery planning, image guided targeting for DBS surgery, postoperative stimulation parameters optimization, and better understanding of the disease and DBS mechanism.

## Supporting information

S1 Table STN trajectory lengths based on MER vs. 7T MRI. This table compares between the length of the STN, as defined by MER at the DBS lead location, and the length of the STN as defined by the 7T images.
(XLSX)

S2 Table STN size and location data. This table provides the full measurements done on the 7T MRI to measure the STN size, shape and location.
(XLSX)
